# Delayed Titanium Hypersensitivity and Retained Foreign Body Causing Late Abdominal Complications

**DOI:** 10.1155/2021/5515401

**Published:** 2021-03-02

**Authors:** Molly S. Jain, Sivasthikka Lingarajah, Enkhmaa Luvsannyam, Manoj Reddy Somagutta, Ravi Pankajbhai Jagani, Jay Sanni, Enomfon Ebose, Frederick M. Tiesenga, Juaquito M. Jorge

**Affiliations:** ^1^Saint James School of Medicine, USA; ^2^Windsor University School of Medicine, Saint Kitts and Nevis; ^3^Avalon University School of Medicine, USA; ^4^California Institute of Behavioral and Neurosciences and Psychology, USA; ^5^All Saints University College of Medicine, Saint Vincent and the Grenadines; ^6^All Saints University School of Medicine, Dominica; ^7^West Suburban Medical Center, USA

## Abstract

Titanium is a known metal used widely in the medical field and can cause allergic reactions with complications. Our case is about a 28-year-old female presenting with a spectrum of abdominal symptoms with a complicated medical history. The abdominal pain is associated with titanium allergy reaction from previously inserted titanium-based surgical clips. This patient is concurrently found to have a retained pigtail catheter in the cecum discovered incidentally through radiology. We discuss the presentation, investigations, and treatment of this unusual case. The case also unfolds rare differential diagnoses to keep in mind when encountering a patient with abdominal pain and associated nonspecific symptoms.

## 1. Introduction

Metal allergies are becoming more recognized within the surgical field. Allergic responses to metals such as nickel, chromium, mercury, cobalt, and gold are widely recognized [1]. Titanium is another type of metal that is utilized extensively in cardiac pacemakers, dental procedures, arthroplasty, body piercing, and spectacle frames [2]. Titanium has also been reported as an allergen in a few studies, causing type I or type IV hypersensitivity reactions [3]. Patients can present with atopic dermatitis, pruritus, edema, urticaria, impaired healing fractures, pain, and necrosis of implants [3]. A case by Little et al. reports an 86-year-old woman who presented three years to postlaparoscopic cholecystectomy with subdiaphragmatic abscess due to titanium surgical clips used during the procedure [4]. Other case reports by Shi et al., Tawil et al., and Towers et al., mentioned hypersensitivity reactions due to titanium in different surgical procedures such as anterior cervical discectomy, dental implants, and spinal fixation devices. However, all the symptoms were resolved after the removal of the offending agent [5–7].

In addition to metal allergies, retained foreign surgical bodies are a potential postoperative complication. Usually retained foreign bodies are found after complaints such as pain, infections and a palpable mass. Infrequent complications have been discussed in studies regarding pigtail catheter found on computerized tomography (CT) scan following a procedure. Pigtail plastic stents are used to remove the intra-abdominal drainage during procedures such as endoscopic retrograde cholangiopancreatography (ERCP) [8].

We describe a case of chronic abdominal pain, which is potentially complicated by retained titanium surgical clips and subsequently a retained portion of a pigtail catheter.

## 2. Case Presentation

We present a case of a 28-year-old female with a chronic history of intermittent abdominal pain aggravated due to titanium allergy and retained foreign surgical body. The patient's past medical history consists of depression, polycythemia vera, hypothyroidism, mitral valve prolapse, chronic back pain, and deep vein thrombosis of nonextremity and acute cholecystitis. The patient's past surgical history includes a tonsillectomy, an appendectomy, and cholecystectomy. The patient's social history is insignificant, with no smoking or alcohol consumption. The patient has allergic symptoms reported from IV contrast, nickel, titanium, iodine, metoclopramide, and oxalate. The patient did not report any screening for immunodeficiency in the past.

**Figure 1:**
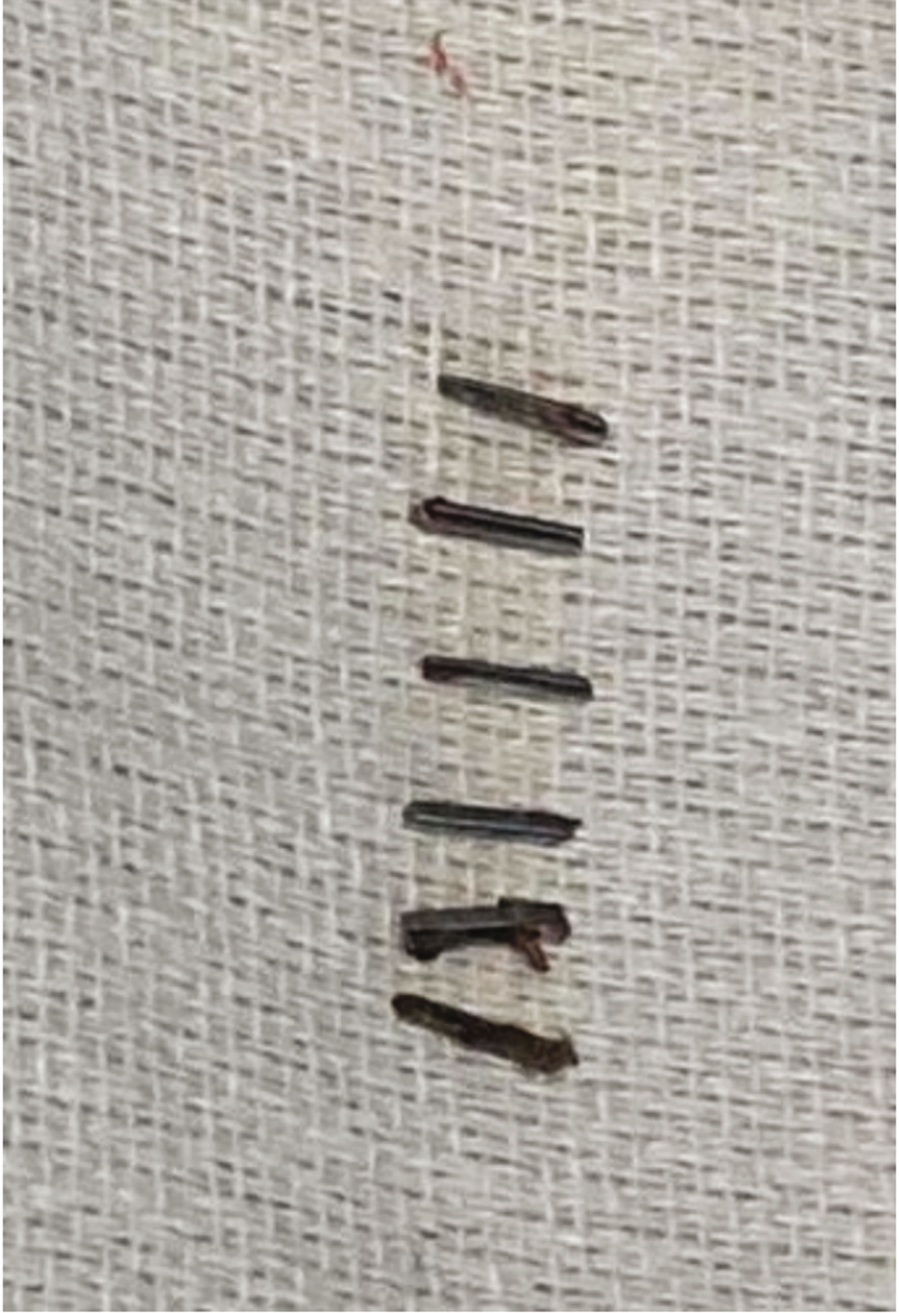
Six metal surgical clips removed from the RUQ via fluoroscopic guidance.

The patient initially presented to the clinic with an intermittent right upper quadrant (RUQ) pain in 2016. She also reported low-grade fever, nausea, vomiting, joint pain, bloody diarrhea, and inability to keep her food down. This resulted in approximately 75 lbs. of weight loss in only four months of duration. Eleven months prior, the patient had undergone cholecystectomy due to acute cholecystitis complicated by a liver abscess. During the cholecystectomy, six titanium surgical clips were placed in the RUQ. The patient had delayed hypersensitivity reaction to nickel in the past with erythematous rash formation and severe itching. With her history of allergy to other metals in the past, a titanium allergy was suspected at this visit, and the patient promptly underwent a titanium skin patch test that turned out positive. To confirm, the patient was further ordered for memory lymphocyte immunostimulation assay (MELISA) that also showed equivocal results for titanium allergy. A decision was made to remove all the metal surgical clips laparoscopically via fluoroscopic guidance (Figure 1). The patient recovered well with no significant intraoperative or postoperative complications. She reported a complete resolution of all symptoms one-month postsurgery.

**Figure 2:**
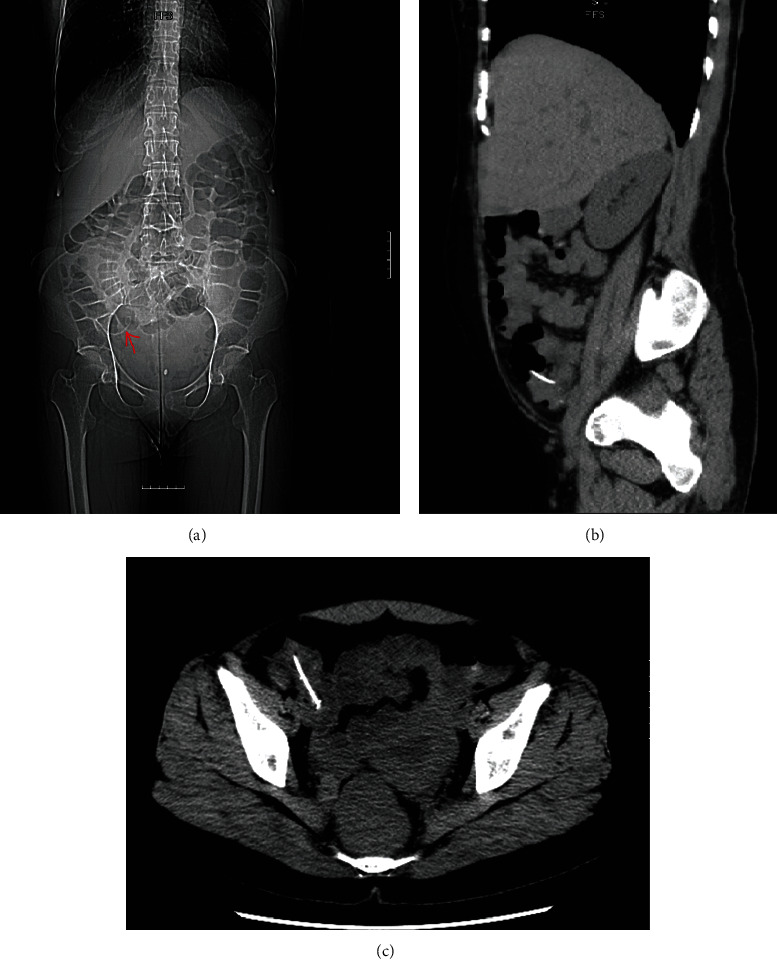
The CT scan showing a portion of the pigtail catheter lodged within the cecum in a coronal view (a), sagittal view (b), and axial view (c).

In the following years, the patient went through an ERCP procedure multiple times due to her sphincter of Oddi dysfunction followed by laparoscopic rectopexy for a rectal prolapse. The patient started having another episode of intermittent right lower quadrant (RLQ) pain starting in January 2020. The pain was sharp and stabbing in nature and radiated from the umbilicus, and the patient rated the pain 10/10 on a scale, causing her significant discomfort. The patient also started having low-grade fever, nausea, vomiting, joint pain, diffuse rash, and fatigue. She was diagnosed with pneumonia twice followed by two sinusitis episodes from January 2020 to March 2020. All of her symptoms were initially suspected due to the infections mentioned above, and she was placed on various antibiotic therapy regimens consisting of ciprofloxacin, amoxicillin/clavulanate potassium, and sulfamethoxazole/trimethoprim. In September 2020, the patient went to a walk-in clinic due to urinary tract infection (UTI) symptoms and was prescribed a course of cephalexin which resolved the infection. However, in October 2020, the patient started having new symptoms of watery, nonbloody diarrhea associated with her chronic RLQ pain. She was tested for *Clostridium difficile* infection and showed positive toxin B results confirming the diagnosis. The patient was referred to an infectious disease specialist and kept on a course of oral vancomycin and fidaxomicin. Within a few days, she started having vomiting, bloating, weakness, fatigue, and intense RLQ abdominal pain. She had to be hospitalized with strict isolation to wait for her medication regimen to work along with the infusion of IV fluids. Her symptoms became chronic and progressed further to abdominal distension with increasing abdominal girth, fever, and several episodes of watery diarrhea. CT scan was promptly ordered to rule out the severe complication of toxic megacolon as a result of *C. difficile* infection. The CT impressions showed no signs of bowel obstruction or colitis; however, it was evident that a portion of a pigtail drainage catheter was lodged within the cecum (Figure 2). The patient contacted our department the next morning, and the case and CT findings were reviewed.

**Figure 3:**
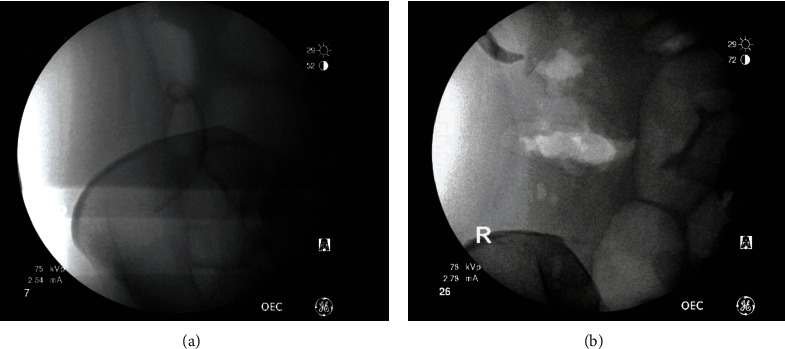
Diagnostic laparoscopy evaluation with fluoroscopic guidance was performed to locate the exact positioning of the pigtail catheter within the cecum before surgery (a) and after pigtail catheter removal (b).

**Figure 4:**
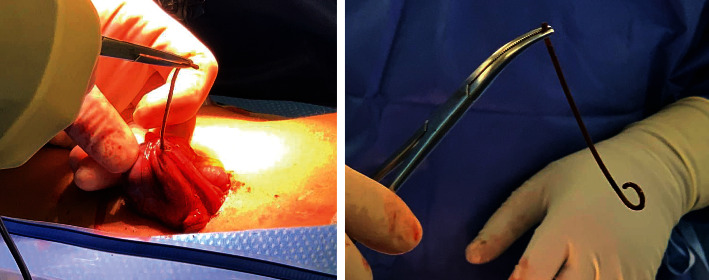
The pigtail catheter in the cecum was removed carefully, and the foreign body was measured 10.0 cm in length and 0.3 cm in diameter.

On November 5, 2020, the embedded right colon foreign body was identified with fluoroscopic guidance (Figures 3(a) and 3(b)) and removed surgically (Figure 4). The patient's preop white blood cell (WBC) count was 5.2 K/*μ*L, absolute neutrophil count was 3281 cells/*μ*L, and absolute lymphocyte count was 1284 cells/*μ*L. The surgical pathology report for the removed catheter specified “foreign body” consisting of synthetic stent measuring 10.0 cm in length and 0.3 cm in diameter (Figure 4). The patient tolerated the procedure well with no postoperative complications. By postoperative day 6, the patient's symptoms of abdominal distension resolved and her RLQ pain also diminished. The patient has been recommended to do a close follow-up for a few months to monitor her progress.

## 3. Discussion

The striking features of titanium include its stability with increased resistance to corrosion and its biocompatibility to diverse materials [3]. This makes it an adequate metal of choice for intraosseous use in medical procedures. Metals including titanium can form haptenic antigens in their ionic form upon bonding with human cell native proteins and have the ability to potentiate degranulation of basophils and mast cells [3]. Type I hypersensitivity reaction may manifest as anaphylaxis causing urticarial plaques or papules on the skin [3]. Moreover, titanium has been described to activate macrophages through phagocytosis to cause the release of pro- and anti-inflammatory cytokines [9]. This causes type IV hypersensitivity reaction through sensitization of T lymphocytes and macrophages. It causes delayed clinical manifestations after 48 to 72 hours [10]. Some symptoms include chronic dermatitis and other reactions depending on the specific organ involved [10].

Diagnosing any metal allergy, including titanium allergy, has been complicated by various tests' sensitivity and specificity. The skin patch test has not yet been developed as the valid standardized test for titanium allergy. Okamura et al. suggested using 0.1% and 0.2% titanium sulfate solution and 0.1% and 0.2% titanium chloride as alternative reagents to titanium oxide in skin-patch testing [11]. However, the skin-patch test is still not the best diagnostic modality due to failure in positive reaction to most titanium allergy cases [3]. The MELISA test has been known to be a more sensitive test to metal allergy but lacks specificity in the proliferation of lymphocytes [3]. LTT is another more sensitive test than the skin-patch test; however, there are reports of some false-positive results [3]. Finally, the blood test could be effectively used to diagnose type IV hypersensitivity allergic reactions, including contact dermatitis or eczema reported by few studies on titanium allergy [12]. The management of any metal allergy including titanium involves removing specific offending agents and avoiding potential triggers to hypersensitivity reactions. In our case, the patient's nonspecific symptoms due to the titanium clips were quite different from the symptoms reported in other studies and may not wholly be attributable to titanium hypersensitivity since the patient had known allergic reactions to various other allergens as well. However, it indeed seems titanium allergy had a significant role in her abdominal discomfort owing to her positive allergy test results further proving titanium hypersensitivity in this patient. Moreover, all her aforementioned symptoms were alleviated once the metal surgical clips were removed surgically.

Retained foreign bodies (RFB) are also possible complications of any surgical procedure. There are more than 28 million surgical procedures with up to 1500 cases of retained foreign bodies estimated in the United States per year [13]. Symptoms can arise as acute inflammatory response, infection, abscess, or aseptic inflammation and exudative without infection in the body cavities [13]. Among different RFB, sponges are one of the most common retained foreign bodies in the abdomen, pelvis, and retroperitoneal cavities [13]. Additionally, standard surgical instruments such as electrodes, drains/catheters, retractors, or clamps can be left unrecognized after operations, especially in the abdominal space [13].

A pigtail catheter is a universal drain used to remove fluids or abscesses from organs, ducts, or body cavities. Some of the reported complications of pigtail catheter insertion include lung penetration during pleural effusion removal and entrapment of the catheter in the tricuspid valve leaflet during heart catheterization [8, 14, 15].

In our case, the pigtail catheter was found as a retained surgical body lodged in the patient's cecum. The patient initially had intermittent RLQ abdominal pain accompanied by nonspecific symptoms. Due to ongoing chronic symptoms, CT scan incidentally revealed a portion of a lodged pigtail catheter in the cecum. The catheter was removed surgically which resolved the patient's RLQ abdominal pain and accompanying symptoms.

It is important to diagnose RFB promptly and remove it as soon as possible due to life-threatening complications such as gastrointestinal bleeding, intestinal obstruction, and perforation [13]. RFB should be included in the differential diagnoses when the patient presents with acute reactions after surgical procedures. This requires immediate attention and an X-ray for further investigation. After the diagnosis of RFB, the preferred approach is to remove the RFB laparoscopically [13]. However, in chronic cases of months to years after the operation, a CT scan is usually performed to rule out malignant tumors and possible fistula formations [13].

It can be challenging to diagnose and discover RFB due to the different manifestations of each case and nonspecific symptoms. Especially, in our case, the patient's extensive past medical history and multiple overlapping medical conditions delayed her diagnosis of RFB and caused her significant life distress. Moreover, the patient was under multiple specialists' care and had many hospitalizations and a history of various medication intake, including antibiotics and painkillers. Regardless of all these factors, surgeons and clinicians must have a high clinical suspicion of RFB and appropriate diagnostic imaging for diagnosis and removal of the RFB. This will improve the patient's quality of life and prevent serious complications.

## 4. Conclusion

This case highlights the variable presentation of titanium allergy that clinicians should be vigilant for. Metals and their alloys could lead to immediate or delayed hypersensitivity reactions. Moreover, metal allergy screening should be considered for patients with past hypersensitivity reactions before any surgery. It also sheds light on retained foreign bodies as a possible cause of abdominal pain in patients who have gone through various surgical procedures in the past. Proper history taking, appropriate physical exams, and specific diagnostic modalities can identify rare causes of chronic abdominal pain.

## Data Availability

No data were used during this study.

## Abbreviations

LTT: Lymphocyte transformation test
MELISA: Memory lymphocyte immunostimulation assay
CT: Computerized tomography
ERCP: Endoscopic retrograde cholangiopancreatography
RUQ: Right upper quadrant
RLQ: Right lower quadrant
UTI: Urinary tract infection
PCR: Polymerase chain reaction
WBC: White blood cell
RFB: Retained foreign body.
